# Supplementary material for: Monolithic back-end-of-line integration of phase change materials into foundry-manufactured silicon photonics
Source: Nat Commun. 2024 Mar 30;15:2786. doi: 10.1038/s41467-024-47206-7 (PMC10981744; doi:10.1038/s41467-024-47206-7)
Supplement: Supplementary file 1 — Supplementary Information [file 41467_2024_47206_MOESM1_ESM.docx]

**Supplementary information for Monolithic back-end-of-line integration of phase change materials into foundry-manufactured silicon photonics**

Maoliang Wei^1,†^, Kai Xu^1,†^, Bo Tang^2,†^, Junying Li^1,3,*^, Yiting Yun^1^, Peng Zhang^2^, Yingchun Wu^4,5^ Kangjian Bao^4,5^, Kunhao Lei^1^, Zequn Chen^4,5^, Hui Ma^1^, Chunlei Sun^4,5^, Ruonan Liu^2^, Ming Li^6,*^, Lan Li^4,5,*^, Hongtao Lin^1,*^

^1^ The State Key Lab of Brain-Machine Intelligence, Key Laboratory of Micro-Nano Electronics and Smart System of Zhejiang Province, College of Information Science and Electronic Engineering, Zhejiang University, Hangzhou 310027, China

2 Institute of Microelectronics of the Chinese Academy of Sciences, Beijing 100029, China

^3^ Hangzhou Institute for Advanced Study, University of Chinese Academy of Sciences, Hangzhou 310024, China

4 Key Laboratory of 3D Micro/Nano Fabrication and Characterization of Zhejiang Province, School of Engineering, Westlake University, Hangzhou, Zhejiang 310030, China

5 Institute of Advanced Technology, Westlake Institute for Advanced Study, Hangzhou, Zhejiang 310024, China

6 State Key Laboratory on Integrated Optoelectronics, Institute of Semiconductors, Chinese Academy of Sciences, Beijing 100083, China

† These authors contributed equally to this work.

* Corresponding author: [junyingli@zju.edu.cn](mailto:junyingli@zju.edu.cn); [ml@semi.ac.cn](mailto:ml@semi.ac.cn); [lilan@westlake.edu.cn](mailto:lilan@westlake.edu.cn); [hometown@zju.edu.cn](mailto:hometown@zju.edu.cn)

SI.1: Performance of the push-pull MZI modulator with the trimming unit


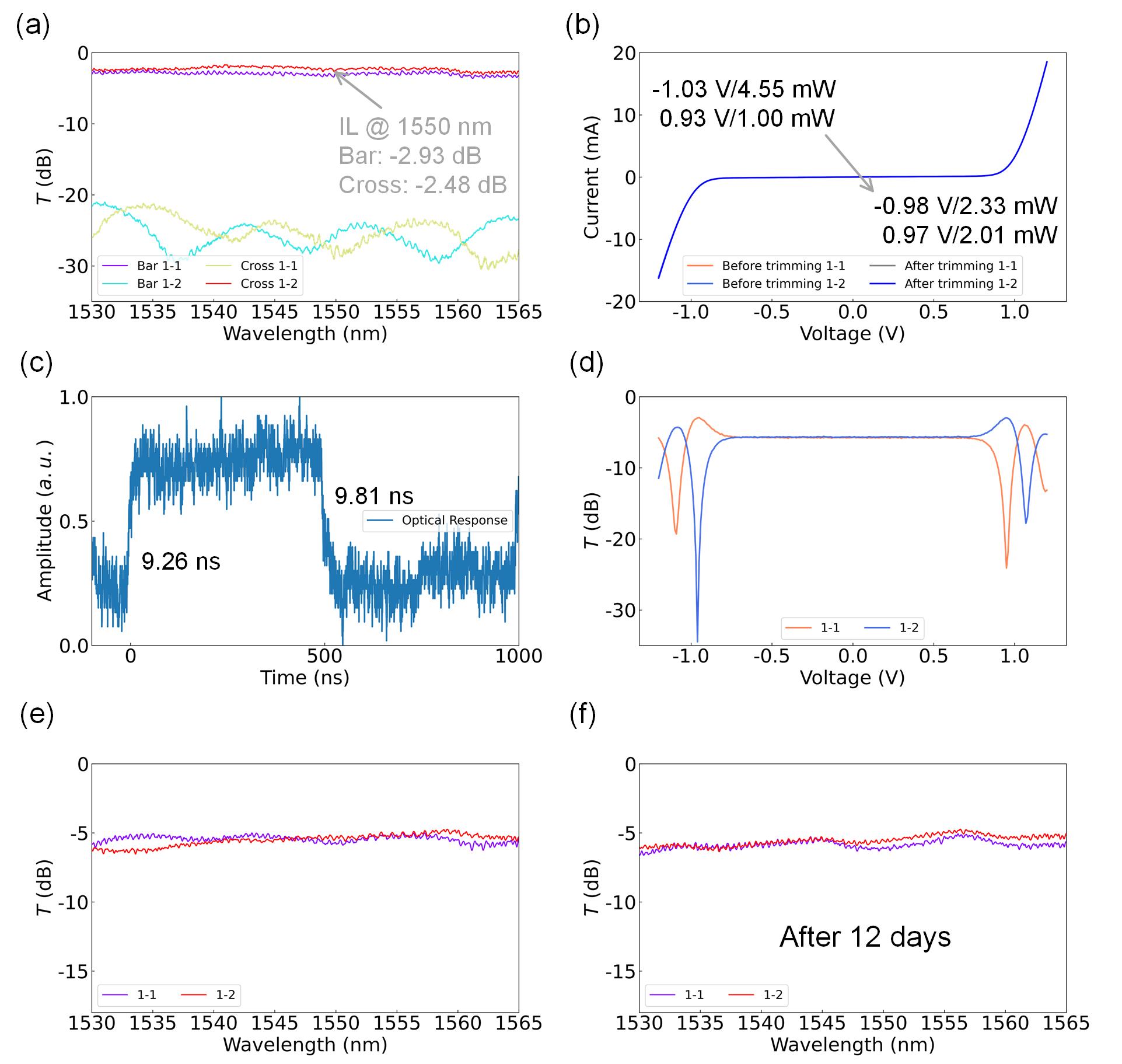


Fig. S1 Optical and electrical performance of the push-pull MZI modulator with the trimming unit. (a) Measured spectra in the bar and cross states before trimming. (b) Measured current-voltage (I-V) curves before and after the trimming process. (c) Measured dynamic response after the trimming process. (d) Measured O-V curves after applying a 1 MHz radio frequency (RF) signal that lasted for 15 minutes. The performance of the device remains stable after the dynamic response measurement. (e) Spectra measured after the RF signal applied. (f) Spectra measured after 12 days, indicating that the post-trimming possesses good stability and a long retention time.

SI.2: Insertion loss of the Sb_2_Se_3_/PIN-doped-Si hybrid waveguide


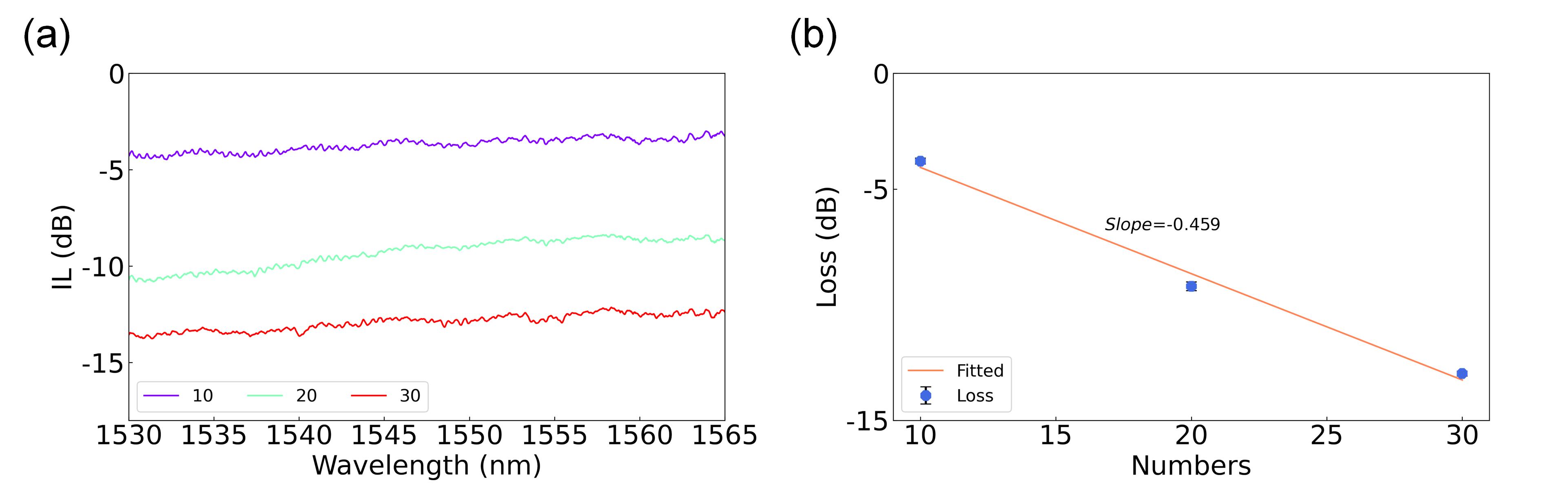


Fig. S2 Loss of the fabricated Sb_2_Se_3_/Si hybrid waveguide. (a) Measured spectra for different numbers of cascaded devices. (b) Extracted loss and linear fit result, indicating that the loss of each Sb_2_Se_3_/Si patch is 0.459 dB. The error bars corresponding to the standard deviation of multiple loss characterization results.

SI.3: Push-pull MZI switch without Sb_2_Se_3_-assisted trimming


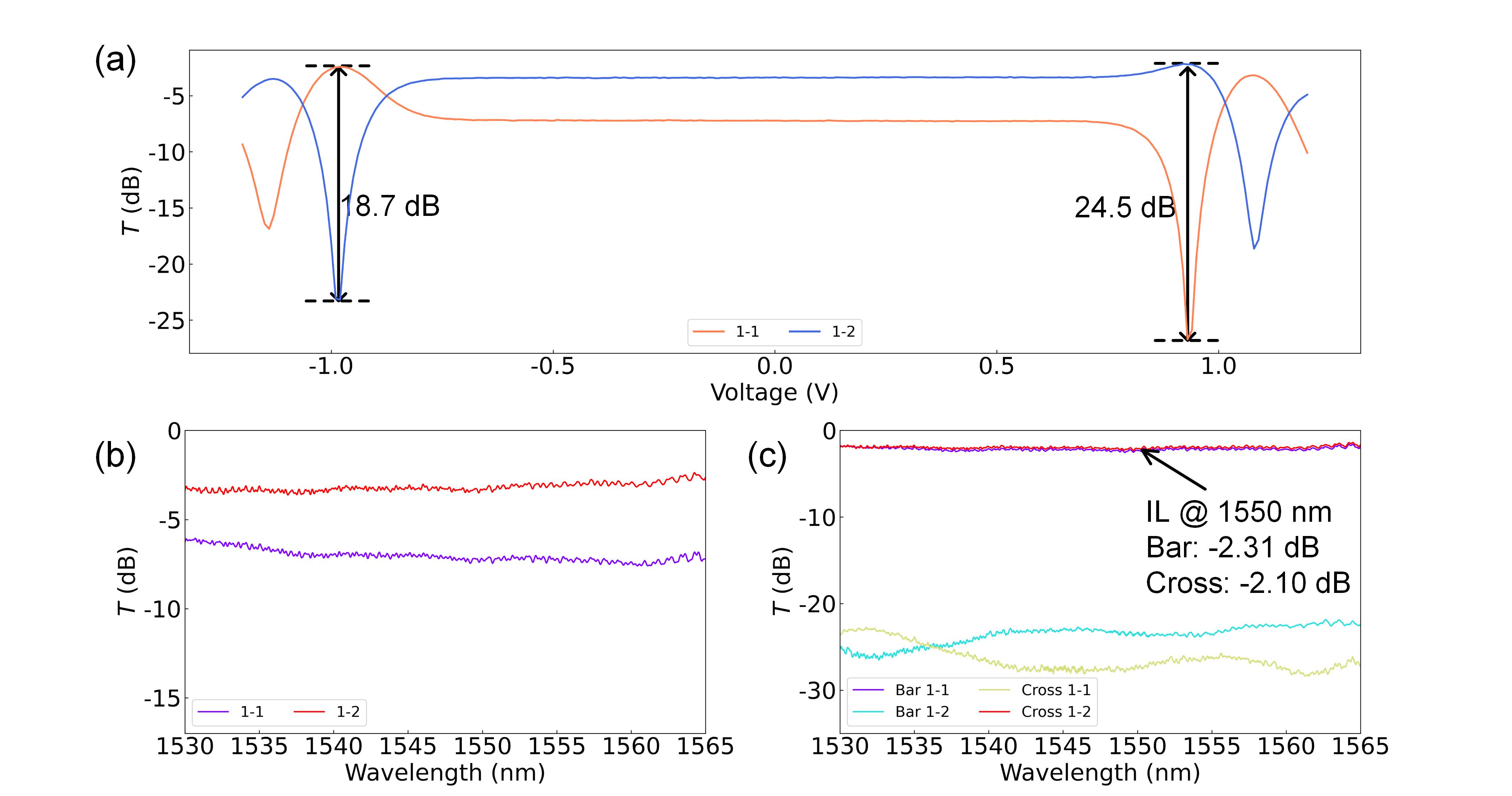


Fig. S3 Performance of the push-pull MZI modulator without Sb_2_Se_3_ deposition. (a) Measured O-V curves. The extinction ratio is 18.7 dB (24.5 dB) for the bar (cross) state. (b) Measured spectra at zero bias. (c) Measured spectra of the MZI modulator revealing an insertion loss of 2.31 dB/2.10 dB in the bar/cross state.

SI.4: Simulated loss due to mode mismatch


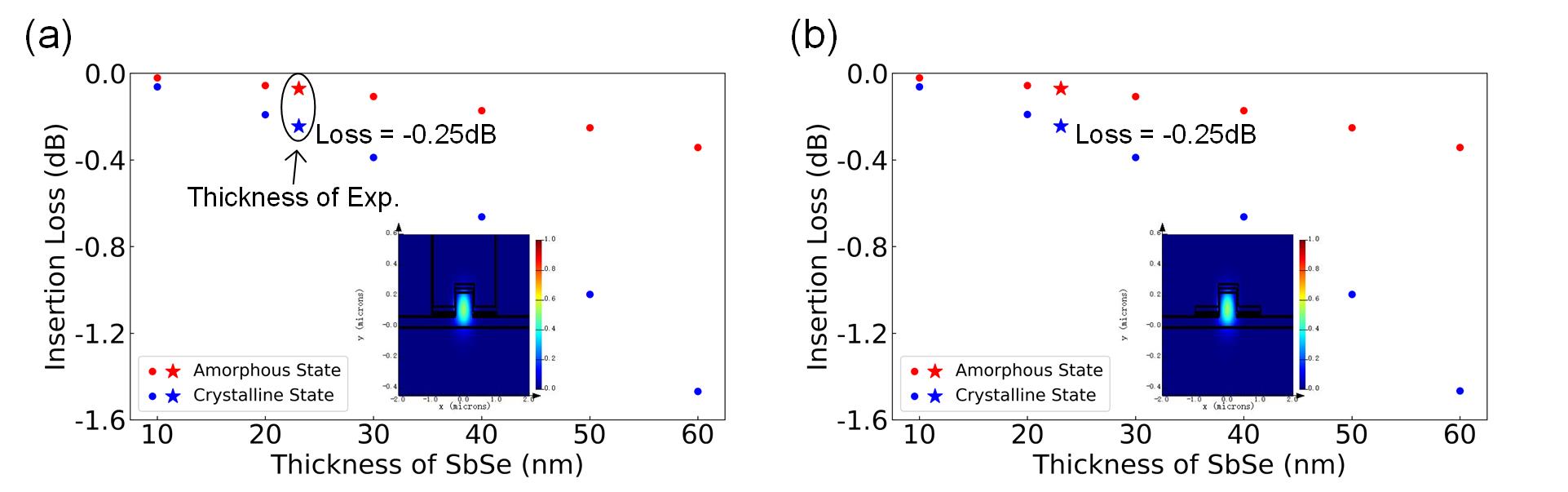


Fig. S4 Simulated loss originating from the mode mismatch at the interface between an Sb_2_Se_3_/Si hybrid waveguide and a silicon waveguide. (a) Simulated mode-mismatch-induced loss in a back-end integrated Sb_2_Se_3_/Si hybrid waveguide. (b) Simulated loss of a typical Sb_2_Se_3_/Si hybrid waveguide. The simulation indicates that the optical loss is mainly due to mode mismatch instead of the SiO_2_ trench on the waveguide.

SI.5: Measured quasi-continuous crystallization process of the Sb_2_Se_3_-assisted MRR switch.





Fig. S5 (a) Measured quasi-continuous crystallization of the phase-modulated MRR switch. The transmittance change ($\Delta T$) was measured at 1551.513 nm.
